# The Epidemic of HIV, Syphilis, Chlamydia and Gonorrhea and the Correlates of Sexual Transmitted Infections among Men Who Have Sex with Men in Jiangsu, China, 2009

**DOI:** 10.1371/journal.pone.0118863

**Published:** 2015-03-16

**Authors:** Geng-Feng Fu, Ning Jiang, Hai-Yang Hu, Tanmay Mahapatra, Yue-Ping Yin, Sanchita Mahapatra, Xiao-Liang Wang, Xiang-Sheng Chen, Giridhar R. Babu, Xiao-Qin Xu, Ping Ding, Tao Qiu, Xiao-Yan Liu, Hongxiong Guo, Xi-Ping Huan, Weiming Tang

**Affiliations:** 1 Jiangsu Provincial Center for Disease Prevention and Control, Nanjing, China; 2 National Center for STD Control, Chinese Center for Disease Control and Prevention, Nanjing, China; 3 Department of Epidemiology, University of California Los Angeles, Los Angeles, California, United States of America; 4 University of North Carolina Project-China, Guangzhou, China; Fudan University, CHINA

## Abstract

**Background:**

In China, the HIV/AIDS epidemic is expanding among men who have sex with men (MSM). As independent risk factors of HIV infection, the epidemics of Chlamydia (CT) and Gonorrhea (NG) in MSM were not well studied, particular for the risk factors of these infectious. The objectives of current reported study were to understand the dynamics of HIV and other sexual transmitted infections (STIs) among MSM in Jiangsu, China, and to measure factors that correlated with STIs.

**Methods:**

In order to gain more participants, a multisite cross-sectional study design was used in our study, by using convenience-sampling to recruit MSM in two Changzhou and Yangzhou, Jiangsu, China, between the July and October of 2009.

**Results:**

In this comprehensive survey involving MSM in two cities of Jiangsu province of China, the prevalence of STIs of CT (6.54%), NG (3.63%), syphilis (20.34%) and HIV (11.62%) were measured. Overall, the STIs prevalence (CT, NG or syphilis) for the participants in our study was 26.39%, meanwhile, 3.4% (14 out of the 413) participants had at least two kinds of STIs. Meeting casual partners at parks, public restrooms or other public areas, having had anal sex with men in the past six months, having had STI symptoms in the past year were positively correlated with STIs positive, with adjusted ORs of 4.61(95%CI 1.03–20.75), 1.91(95%CI 1.14–3.21) and 2.36(95%CI 1.07,5.24).

**Conclusion:**

Our study findings reiterate the fact that Chinese MSM are highly susceptible to acquiring syphilis, CT, NG and HIV, and there is an urgent need for intervention targeted towards this population. Behavioral measures should constitute an important part of the targeted intervention. Furthermore, the already implemented preventive and diagnostic services for HIV should be expanded to include syphilis CT and NG, too.

## Introduction

In China, the HIV/AIDS epidemic is expanding among MSM. This was demonstrated by the increasing proportion of persons living with HIV and AIDS and number of newly diagnosed HIV infections among Men who have Sex with Men (MSM)[1]. This expanding epidemic of HIV/AIDS among MSM is considered as one of the biggest public health concerns in China[2]. In addition, the expanding epidemics of Sexually Transmitted Infections (STIs), namely syphilis, Chlamydia (CT) and Gonorrhea (NG) in MSM is alarmingly increasing. Previous studies have demonstrated that the presence of any of these infections could potentially increase the risk of acquiring HIV and other STIs[3] which in turn can worsen the current scenario among MSM in China [4].

Jiangsu, a coastal province in eastern China, also experienced the similar rise in epidemic of HIV and other STIs[5,6]. This problem is more serious in Changzhou and Yangzhou, which were the study sites of the current designed study[7]. However, only few studies have reported the burden of CT and NG infections and their correlates in MSM. Also, there is limited evidence regarding current epidemiological situation of syphilis and HIV in MSM in Jiangsu province. The high burden of HIV/STIs in Yangzhou and Changzhou require imminent interventions for effective control. However, all the intervention programs implemented in these places in MSM until now were based on the successful experiments in the other regions. Thus, a comprehensive survey was designed to know the dynamics of HIV and STIs epidemic and the factors correlated with this epidemic in MSM in Jiangsu. This study can identify the gaps and guide the policy-makers in designing intervention programs specifically targeted towards the need for control of these epidemics among MSM.

The objectives of this study were:

1. To understand the dynamics of HIV and STIs epidemic in Yangzhou and Changzhou.
2. To measure the association of STIs with their potential correlate factors like HIV status, behaviors and other factors.

## Materials and Methods

### Study participants enrolment

In order to gain more participants, a multisite cross-sectional study design was used in our study, by using convenience-sampling to recruit MSM in two Changzhou and Yangzhou, Jiangsu, China, between the July and October of 2009. The participants were recruited at venues where the participants met their sexual partners and STIs clinics. In addition, we also put banners on gay website platforms and venues, to motivate the participants come to the study sites to attend the survey.

To meet the inclusion criteria, the participants had to be male, had sex with men (oral and/or anal) within the past year, aged 18 years or older and were willing to participate voluntarily. In addition, the participants were excluded from the study if they had medical reasons or intoxication preventing them from active participation in the study, currently or previously enrolled in any other HIV behavioral intervention trial in the past 3 months.

### Structured Interview

Written informed consent was collected from the eligible participants followed by collection of the biological samples and a face-to-face interview with a structured questionnaire for information regarding socio-demographic characteristics, HIV knowledge, recent sexual behaviors (with male), drug use, and sex behaviors with female partners. Demographic information included age (categorized into less than 25, 25 to 40, older than 40), marital status (single, married and divorced/widowed), education level (elementary school or lower, junior or senior high school, college or higher), and residency (“hukou” status: official resident of Yangzhou and Changzhou cities, other cities in Jiangsu province or other provinces) were also collected in our study. In addition, cruising areas/venues in our study were categorized as gay bars, parks, massage parlors, spas, saunas, internet and others (such as met partners in college campuses, introduced by friends etc). Recent sexual behavior included whether engaged in anal sex (with male) and vaginal sex, whether condom was used during the last anal (with casual or regular partners) and vaginal intercourse.

The test results for HIV, CT, NG and syphilis were combined later with the data collected during face-to-face interviews using the structured questionnaire. Participants diagnosed positive for the Western Blot test for HIV antibody were defined as HIV positive while persons positive for both Toluidine Red Unheated Serum Test (TRUST: A Qualitative and Quantitative Card Test for the Serologic Detection of Syphilis) and ELISA were defined as Syphilis positive. Participants diagnosed positive for the PCR test for CT or NG were defined as CT or NG positive. Participants diagnosed positive for CT, NG or syphilis were defined as STIs positive; or else, the participants were defined as STIs negative.

Participants who were HIV positive were referred to National HIV care and treatment program for the further follow up and treatment. And those who were positive for STIs (syphilis, CT or NG) were counseled, and were referred to designated clinics or disease control centers for further evaluation and possible treatment.

### Biological measures

Five ml of venous blood was collected from each participant for HIV and syphilis testing. HIV antibodies were screened using a rapid test (Acon Biotech Co., Ltd). If the screening result was positive, it was confirmed by Western Blot (HIVBLOT 2.2, Genelabs Diagnostics, Singapore). In order to reduce the potential for false negative HIV cases due to the window period, HIV Nucleic Acid Amplification Testing (NAAT)was done for every rapid tested sero-negative participant at each round of the study. Syphilis antibodies were screened using ELISA (Wantai Biopharmacy Co., Ltd) test and confirmed with TRUST (Wantai Biopharmacy Co., Ltd).

Urine sample was also collected from each participant. The urine samples were evaluated at the National STD Reference Laboratory at Nanjing for NG and CT testing by using Polymerase Chain Reaction (PCR) (Roche Amplicor assay, Roche Diagnostic Systems, Indianapolis, IN).

### Data Analysis

Data were double-entered and checked using EpiData 3.0.1. After the cleaning of the data, SAS statistical analysis software version 9.1 (SAS INSTITUTE INC.) was used to conduct the descriptive analyses of demographics and behavior of the participants. In addition, we used logistic regression to perform univariate analysis and multivariate analysis to test the factors correlated with STIs.

### Ethical statement

The study process and content were approved by the Ethics Committee of Jiangsu Provincial Center for Disease Prevention and Control (JSCDC). Informed consent was obtained from each of the participants prior to biological samples collection and the interviews. Each of the participants had the freedom to decline or to withdraw from this survey at any time freely.

## Results

### Demographic and sexual behaviors

In our study, 413 MSM were enrolled. Among these participants, 123 participants (29.78%) accepted the survey at Yangzhou while the other 290 (70.22%) participants attended the survey at Changzhou. Table 1 shown that about one quarter of the participants were aged less than 25 years, and about two thirds of them only attended junior or senior high school. About half of the participants (52.30%) were married, and about 44.55% of them were living with female partners. In addition, about 46.73% of the participants were residents of the sampled cities, and about 68.52% of the participants defined themselves as homosexuals. About 43.34% of the participants found their casual partners at bathhouse. Among the participants, about two third of them engaged in anal sex with other men in the past six months, meanwhile, about 54.48% of them had sex with female in the past three months.

**Table 1:** Demographics, sexual behavior and syphilis prevalence among recruited MSM in the Yangzhou and Changzhou cities of Jiangsu, China, 2009 (N = 413).

Variable		STIs = 0 (N = 304)			STIs = 1 (N = 109)			Overall (N = 413)	
		Frequency	Percent	95%CI	Frequency	Percent	95%CI	Frequency	Percent
Age	Less than 25	80	26.32	21.34,31.29	22	20.18	12.53,27.84	102	24.70
	25 to 40	143	47.04	41.40,52.68	54	49.54	40.00,59.08	197	47.70
	More than 40	81	26.64	21.65,31.64	33	30.28	21.51,39.04	114	27.60
Education	Illiterate or elementary school	9	2.96	1.04,4.88	7	6.42	1.75,11.10	16	3.87
	Junior High school or senior high school	196	64.47	59.06,69.88	78	71.56	62.96,80.16	274	66.34
	College or above	99	32.56	27.27,37.86	24	22.02	14.11,29.92	123	29.78
Marital	Single	133	43.75	38.14,49.36	39	35.78	26.64,44.92	172	41.65
	Married	155	50.99	45.34,56.64	61	55.96	46.49,65.43	216	52.30
	Divorced or widowed	16	5.26	2.74,7.79	9	8.26	3.01,13.51	25	6.05
Living	Living alone	110	36.18	30.75,41.62	40	36.70	27.50,45.89	150	36.32
	With female partner	133	43.75	38.14,49.36	51	46.79	37.27,56.31	184	44.55
	With male partner	27	8.88	5.66,12.08	6	5.50	1.15,9.85	33	7.99
	Other people	34	11.18	7.62,14.75	12	11.01	5.04,16.98	46	11.14
Resident	Sampling city	140	46.05	40.42,51.69	53	48.62	39.09,58.16	193	46.73
	Other cities in Jiangsu province	90	29.61	24.44,34.77	27	24.77	16.54,33.00	117	28.33
	Other Provinces	74	24.34	19.49,29.19	29	26.61	18.18,35.03	103	24.94
Sex orientation	Homosexual	214	70.39	65.23,75.56	69	63.30	54.11,72.50	283	68.52
	Bisexual	90	29.61	24.44,34.77	40	36.70	27.50,45.89	130	31.48
Main venues for seeking male sexual partners	Pub, disco, tearoom, or club	91	30.95	25.64,36.27	28	25.93	17.53,34.32	119	28.81
	Spa, bathhouse, sauna, massage	128	43.54	37.84,49.24	51	47.22	37.65,56.79	179	43.34
	Internet	47	15.99	11.77,20.20	20	18.52	11.07,25.96	67	16.22
	Park, public restroom, or other public areas	3	1.02	0.00,2.18	5	4.63	0.60,8.66	8	1.94
	Others	25	8.50	5.30,11.71	4	3.70	0.08,7.32	29	7.02
Had anal sex with other men in the past six months	Yes	197	64.80	59.40,70.20	81	74.31	65.98,82.64	278	67.31
	No	107	35.20	29.80,40.60	28	25.69	17.35,34.02	135	32.69
Had sex with female in the past six months	Yes	158	51.97	46.32,57.62	67	61.47	52.18,70.75	225	54.48
	No	146	48.03	42.38,53.67	42	38.53	29.25,47.81	188	45.52
HIV	Negative	274	90.13	86.76,93.50	91	83.49	76.40,90.57	365	88.38
	Positive	30	9.87	6.50,13.24	18	16.51	9.43,23.60	48	11.62
Study sites	Yangzhou	74	24.34	19.49,29.19	49	44.96	35.47,54.44	123	29.78
	Changzhou	230	75.66	70.81,80.51	60	55.04	45.56,64.53	290	70.22
CT	Positive	0	0.00	——	27	24.77	16.54, 33.00	27	6.54
	Negative	304	100.00	——	82	75.23	67.00,83.46	386	93.46
NG	Positive	0	0.00	——	15	13.76	7.19, 23.23	15	3.63
	Negative	304	100.00	——	94	86.24	76.67,92.81	398	96.37
Syphilis	Positive	0	0.00	——	84	77.06	69.04,85.08	84	20.34
	Negative	304	100.00	——	25	22.94	14.92,30.96	329	79.66

### HIV and STIs Prevalence

In our study, 48 participants were tested HIV positive (none rapid tested sero-negative participant was positive for HIV NAAT test), with an overall HIV prevalence of 11.62%. The prevalence of syphilis was 20.34% (84 participants). In addition, CT and NG prevalence was 6.54% and 3.63% respectively. Overall, 26.39% of participants were defined as STI positive. In the study population, 109 participants were STI positive, of which 95 had only one of the three STIs, 5 had both CT and Syphilis, 3 had both NG and Syphilis and 3 were positive for all three STIs. Participants who had STIs were different in terms of demographic and behavioral factors from those who were STI negative as evident from the non-overlapping CIs presented in Table 1.

Univariate analysis (Table 2) indicated that compared to pubs, discos, tearooms, or clubs, STIs positively correlate with meeting casual partners at parks, public restrooms or other public areas with OR of 5.42 (95%CI 1.22–24.10).

**Table 2:** Predictors for STIs among MSM in Jiangsu, China, 2009 (N = 413).

Variable		Crude		Model 1*		Model 2^**@**^	
		OR	95%CI	OR	95%CI	OR	95%CI
Sexual Orientation	Homosexual	Ref		Ref		Ref	
	Heterosexual	1.38	0.87,2.18	1.30	0.81,2.08	1.29	0.80,2.08
Resident	Sampled city	Ref		Ref		Ref	
	Other cities in Jiangsu Province	0.79	0.46,1.35	0.81	0.47,1.39	0.79	0.46,1.36
	Other provinces in China	1.04	0.61,1.76	1.12	0.63,1.97	1.12	0.62,2.02
Main venues for seeking male sexual partners	Pub, disco, tearoom, or club	Ref		Ref		Ref	
	Spa, bathhouse, sauna, massage	1.30	0.76,2.21	1.07	0.61,1.88	1.08	0.60,1.96
	Internet	1.38	0.70,2.71	1.83	0.89,3.78	1.91	0.92,3.98
	Park, public restroom, or other public areas	5.42	1.22,24.10	4.61	1.03,20.75	4.52	0.99,20.63
	Others	0.52	0.17,1.62	0.53	0.17,1.65	0.56	0.18,1.80
Had anal with men in the past six months	No	Ref		Ref		Ref	
	Yes	1.57	0.96,2.56	1.91	1.14,3.21	1.91	1.14,3.22
Did not use condom in the last intercourse	No	Ref		Ref		Ref	
	Yes	0.55	0.22,1.37	0.60	0.24,1.48	0.55	0.22,1.40
Had sex with female in the past six months	No	Ref		Ref		Ref	
	Yes	1.47	0.94,2.30	1.36	0.86,2.15	1.58	0.93,2.67
Did not use condom in the last vaginal-intercourse	No	Ref		Ref		Ref	
	Yes	0.65	0.35,1.23	0.60	0.32,1.14	0.59	0.30,1.14
HIV	Negative	Ref		Ref		Ref	
	Positive	1.81	0.96,3.40	1.67	0.88,3.18	1.69	0.88,3.22
Had STI symptoms in the past year	No	Ref		Ref		Ref	
	Yes	2.23	1.02,4.87	2.36	1.07,5.24	2.38	1.06,5.3

*Model 1 adjusted for age and education;

^@^:Model 2 adjusted for age, education, city, marital status and living status.

### Factors correlated with STIs

After adjusting for age, education, meeting casual partners at parks, public restrooms or other public areas, having had anal sex with men in the past six months, having had STI symptoms in the past year were still positively correlated with STIs positive, with ORs of 4.61(95%CI 1.03–20.75), 1.91(95%CI 1.14–3.21) and 2.36(95%CI 1.07,5.24), respectively. Even though not statistically significant, compared to reference groups, those who had anal sex with men in the past six months, those who had sex with female in the past six months and HIV positive were highly related to STI positive, with ORs of 1.57(95%CI 0.96–2.56), 1.47(95%CI 0.94–2.30) and 1.81(95%CI 0.96–3.40), respectively. Similar results were found after further adjustments for city, marital status and living status.

## Discussion

In this comprehensive survey involving a representative sample of MSM in two cities of Jiangsu province of China, the prevalence of STIs of CT (6.54%), NG (3.63%), syphilis (20.34%) and HIV (11.62%) were measured. Overall, the STIs prevalence (CT, NG or syphilis) for the participants in our study was 26.39%.

The overall HIV prevalence of these two cities was about 11.62%. Compared to other cities in Jiangsu[8], the HIV and syphilis prevalence among the participants in the two cities was very high, but similar to the HIV prevalence reported by Chongqing [9,10]. The HIV prevalence in our study was higher than that in Beijing[11], Shenzhen[12], Chengdu[13], and other cities in China[14,15]. The syphilis prevalence among MSM participants in our study was about 20%. Which was much higher than one previous observation among MSM in Jiangsu[6] and similar to that in Guangdong and Hebei, but higher than in several other places of China[16]. The high HIV and syphilis prevalence in the two study cities of Jiangsu, China was a warning signal which without effective intervention strategies may bring in a serious burden of HIV/syphilis, and finally may become a critically public health and social problem[2].

The observed proportion for CT was lower than the findings from studies conducted among MSM in Jiangsu China in 2003, however, the NG prevalence was higher than the findings of the study[6]. Also, the CT and prevalence rates in our study was higher than the findings of the previous studies conducted in Nanjing[17], Hongkong[18]. CT and NG prevalence measured in this study was higher than the observations in Vietnam[19], but lower than that in India[20]. Being one of investigations that focused on the burden of CT and NG in Jiangsu based on a representative sample, the observed proportions may be considered as realistic pictures of these STI epidemics in this province necessitating urgent target-oriented disease control programs in MSM.

In our study, 3.4% (14 out of the 413) participants had at least two kinds of STIs. Such a high co-infection proportion was a warning signal, as this co-infection could facilitate the disease processes of each other, and could facilitate the transmission and acquisition of other STIs, including human immunodeficiency virus (HIV)[21].

About three quarters of the recruited MSM were aged less than 40 years and only about 30% had college-level or higher education, while about two thirds of them engaged in anal sex in the past six months. Lack of awareness may translate this lower educational attainment into increased risk of acquisition of STIs including HIV when engaging in risky sexual behaviors[22].

There is possibility that HIV and other STIs might spread into general population. This possibility arises as more than half of the participants were married, about 45% of them were living with their female patterns and about 55% of the participants had sex with women in the past six months. Therefore, urgent attention is warranted to prevent the HIV and STIs transmission from MSM to general population in this part of China [22,23]. In our study, about half MSM were the official residents of the sampling city, which probably meant that half of this population was migrated and thus more likely to have risky sexual behaviors and roll out of disease control programs to them might be difficult[8]. In addition, about 70% of the participants identified themselves as homosexual, about 29% of the participants mainly found their partners at pubs, disco, tearoom, or club, and about 43% of them mainly found their partners at spa, bathhouse, sauna, massage. Considering the evidences from contemporary scientific literature, these demographic and sexual behavioral patterns among MSM of Jiangsu are likely to emphasize the vulnerability of this population regarding acquisition of HIV and other STIs[24].

While estimating the magnitude and direction of association between STIs with their potential correlates, MSM who usually meet their partners at park, public restroom, or other public areas seemed to be more vulnerable for STIs acquisition. This finding was parallel with one previous study conducted at Guangzhou[25]. One possible explanation for this phenomenon was that MSM who went to these venues are younger, less educated, poorer and were more likely to engaging in risk behavior than men who did not go to these venues[26]. Our study indicated that men who had anal sex with men in the past six months were positively correlated with STIs. The potential reason for this scenario is that anal intercourse may cause trauma to the anal mucosa and thus increase the probability of transmitting the virus, particularly when condom was not used during sexual intercourse[8].

Our study also found that those who had STI symptoms in the past year were positively associated with STIs positive. Reverse causation could be one potential reason for this phenomenon. As a cross-sectional study design at baseline, our study had the problem of time ambiguity, and we must admit, it is difficult to draw an inference on the direction of this association based on a cross-sectional data like ours.

Due to the small sample size of HIV positive patients, our study lacked the power to detect the relationship between HIV positive and STIs. Despite of this problem, we still found that HIV was potentially positive with STIs positive, which was parallel with several studies conducted before [27–29]. However, due to the cross-sectional study design, we do not know the direction between the two variables.

The strengths of our study include the use of multiple biomarkers for the participants and recruiting samples from two cities. Being an observational study, our results suffered from quite a few limitations. First, because of our cross-sectional design, lack of temporality prevents us from drawing any causal inferences. The time orders of predictors were often unclear such as whether HIV infection affected disease prevalence or the other way round. Second, the fact that most of our data were self-reported raises concern about social desirability bias that, in turn, may lead to severe exposure misclassification. Third, selection bias, due to the non-response of the participants, can also be a serious threat to validity. Forth, several STIs (syphilis, CT and NG) were combined together as the outcome of our study. Such combination may introduce potential bias as the infection mechanisms of these several diseases were different. Fifth, due to the convenience sampling strategy, the participants in our study could not represent all MSM in these two study cities. Last but not least, only urine samples were collected for CT and NG testing, while throat swabs and anal swabs samples were also usually used for CT and NG testing. Our study may underestimate the burden of CT and NG among MSM in Jiangsu province.

To minimize bias due to data collection, a uniform protocol was followed in every study site, and all the interviewers were trained together by the same program. In addition, HIV, syphilis, CT and NG results were declared to the participants only after the interview was completed. Therefore, we can expect the misclassification of exposure in our study to be mostly non-differential. However, we do not rule out possibility of differential misclassification, because some participants might have been symptomatic or were aware of their disease status before they participated in the study.

Our study findings reiterate the fact that Chinese MSM are highly susceptible to acquiring syphilis, CT, NG and HIV, and there is an urgent need for intervention targeted towards this population. Behavioral measures should constitute an important part of the targeted intervention. Furthermore, the already implemented preventive and diagnostic services for HIV should be expanded to include syphilis CT and NG, too. Finally, a large-scale longitudinal study could be planned to further explore variables associated with HIV, syphilis, CT, NG and other STIs among MSM.

## Supporting Information

S1 Dataset Dataset of the original research. (XLS)
